# Shared Relationship Efficacy of Dyad Can Increase Life Satisfaction in Close Relationships: Multilevel Study

**DOI:** 10.1371/journal.pone.0159822

**Published:** 2016-07-20

**Authors:** Ryosuke Asano, Kenichi Ito, Toshikazu Yoshida

**Affiliations:** 1 Department of Literature, Kurume University, Kurume, Japan; 2 Division of Psychology, Nanyang Technological University, Singapore, Singapore; 3 Institute on Asian Consumer Insight, Singapore, Singapore; 4 Department of Education, Gifu Shotoku Gakuen University, Gifu, Japan; University of Lethbridge, CANADA

## Abstract

Characteristics of relationship itself play an important role in determining well-being of individuals who participate in the relationship. We used efficacy expectations mutually shared between close friends or romantic partners as a characteristic of relationship and investigated its impact on their life satisfaction. In Study 1, we conducted a cross-sectional study among 137 pairs of close same-sex friends to test whether the efficacy expectations shared between friends are associated with levels of life satisfaction. In Study 2, we conducted a longitudinal study among 114 heterosexual romantic couples to test predictive validity of the efficacy expectations shared between couples predict levels of life satisfaction 2 month later. In both studies we found a consistent result that as degrees of the efficacy expectations shared between individuals in a relationship increased, the degree of their life satisfaction also increased. Underlying mechanisms that explain how characteristics of relationship itself increase life satisfaction are discussed.

## Introduction

Establishing an intimate, stable, and meaningful relationship with a friend or a significant other is truly important in life. In order to form, develop, and maintain a good relationship, two individuals must accept their counterpart [1,2], discuss their positive and negative life events [3,4], and contribute their resources for the welfare of the relationship [5,6]. Through these processes, the relationship becomes unique and one-and-only for the two individuals [7,8]. In such intimate relationships, individuals sometimes implicitly share expectations about their joint ability to maintain the relationship. For example, best friends mutually expect that they can prevent undesirable events from occurring, or romantic partners expect that they can reach mutually satisfying compromises during a conflict. These shared efficacy expectations are viewed as unique characteristics of the relationship itself that may not resemble characteristics of each individual. The idea that the relationship itself acquires characteristics not deducible to each partner is not new in the area of close relationship research (e.g., [9]). However, empirical investigation of this idea has been neglected despite the calls for study by prominent researchers (e.g., [10]). In order to fill this gap, we investigated the two individuals’ implicitly shared beliefs about joint ability to coordinate and cooperate with each other to maintain a good relationship [11,12]. In particular, we tested whether dyads’ shared efficacy beliefs in joint ability predicts levels of life satisfaction.

### Efficacy Expectations of Dyad

According to the social cognitive theory [13,14], people successfully achieve goals in their lives when they believe in their abilities to organize and execute strategic plans of action to attain desired outcomes. These efficacy expectations with regard to creating and completing necessary actions to accomplish a goal are called *self-efficacy*, which has received research attention not only in psychology but also in economics, sociology, and medicine. However, in the context of interpersonal relationships that involve two individuals to accomplish a goal (e.g., forming, developing, or maintaining a close relationship), one individual’s belief about his or her own ability is insufficient to explain relationship outcomes.

Past research indicates that efficacy expectations about a relationship can be conceptualized at three distinct levels [15]. The first level involves individuals’ idiosyncratic expectations about their own ability to carry out the behaviors needed to produce the desired outcomes in a dyadic relationship. For example, a boyfriend who believes that he can singlehandedly solve a crisis in his romantic relationship would have a high *self-efficacy of dyad*. This level represents Bandura’s [13,16] initial proposition about self-efficacy beliefs. The second level, although it is also concerned with individuals’ idiosyncratic expectations about ability to create and maintain a good relationship, involves one’s expectations about two people’s joint ability. For example, a boyfriend who believes that he and his romantic partner together possess the ability to solve crisis in their romantic relationship would have a high *idiosyncratic relationship efficacy of dyad*. Murray and Holmes [17] argued that idiosyncratic relationship efficacy of dyad is a benchmark for positive close relationships. The third level, a main target of the current study, is the implicitly shared efficacy expectations between two individuals about their joint ability to organize and execute the actions necessary for achieving the desired outcomes in a relationship. Efficacy expectations at this level develop through an “interactive, coordinated, and synergetic dynamics of transaction” ([14], p.14) and best conceptualized as an emergent property of dyadic relationship rather than individuals’ idiosyncratic beliefs. In combination with shared intentions, knowledge, and skills, it allows partners to achieve desired outcomes beyond what the sum of two people can achieve. For example, a pair of individuals who share similar beliefs in their joint ability to solve a crisis in their romantic relationship would also have a high efficacy of dyad, but this level is distinct from the other levels to the extent that efficacy expectations are characteristics of the relationship itself, rather than of each individual. Throughout this paper, we use the term *shared relationship efficacy of dyad* to distinguish this third level from other levels, and to reflect two people’s shared beliefs about joint ability.

In the previous studies on efficacy expectations of dyad, researchers investigated either self-efficacy of dyad or idiosyncratic relationship efficacy of dyad. For example, Doherty [18] proposed that a spouse would increase effort on problem solving in the marital relationship when he or she felt high self-efficacy of dyad as a result of attributing the partner’s negative behavior to external situations. In contrast, the effort spent on problem solving would be decreased when he or she felt low self-efficacy of dyad and attributed the negative behavior to stable internal characteristics. Fincham et al. [19] extended this line of research by showing that higher levels of self-efficacy of dyad led to an increase in marital satisfaction. For example, a spouse who attributed a partner’s negative behavior to stable internal characteristics (e.g., childhood experiences or personality traits) would feel low self-efficacy of dyad, and as a result, would experience lower marital satisfaction. Instead of self-efficacy of dyad, Murray and Holmes [17], in their study on positive illusion in romantic and marital relationships, focused on an individual’s efficacy expectations of joint ability, or idiosyncratic relationship efficacy of dyad, in his or her relationship. Murray and Holmes asked couples to report, without discussing their answers with their partner, their own idiosyncratic relationship efficacy and typical couples’ idiosyncratic relationship efficacy of dyad. Higher ratings for a participant’s own relationship compared to ratings for a typical romantic relationship indicated positive illusion of idiosyncratic relationship efficacy of dyad. The results showed that a positive illusion predicted greater satisfaction, trust, and longitudinal stability in the relationship.

### Shared Relationship Efficacy of Dyad and Life Satisfaction

Although informative, these studies focused only on each partner’s idiosyncratic efficacy expectations of dyad. Capturing the shared relationship efficacy of dyad as a characteristic of the relationship itself has theoretical and methodological advantages. The literature on close relationships shows that dyad-level characteristics are as important as, if not more important than, individual-level characteristics. Lewin [9] posited that the interdependence between two individuals creates unique characteristics of their relationship, which may not be deducible to the characteristics of each individual. Similarly, Berscheid [10] pointed out that the characteristics of a relationship may not be observable in the two individuals’ mind or psyche, because they are embedded in the dynamic interactions between two persons. Inspired by these arguments, relationship scientists proposed characteristics unique to a relationship itself, such as the agreement of psychological characteristics [20], the shared interpretation of common experiences [21], or the unique pattern of interconnection between persons [22]. Despite these theoretical developments, empirical research on the characteristics of the relationship itself is still scarce (see [23,24], for notable exceptions). The current study sheds light on the shared relationship efficacy of dyad to provide evidence for the effect of unique characteristics of a relationship.

Including dyad-level characteristics, in addition to individual-level characteristics, as predictors would be expected to result in greater predictive validity than that achieved by previous studies on close relationships. Supportive evidence comes from the study of *collective efficacy*, which refers to shared efficacy expectations in joint abilities as a group [13,14]. The only difference between shared relationship efficacy of dyad and collective efficacy is the number of people within the unit; thus, some findings on collective efficacy of group are informative to the current study. In an interesting study on the performance of university basketball teams in the United States, for example, researchers measured individuals’ self-efficacy of group and teams’ collective efficacy of group prior to the beginning of the season. Self-efficacy of group was measured by recording players’ agreement with statements such as “I have very high confidence in my ability to play my position or positions,” while collective efficacy of group was measured by recording players’ agreement with statements such as “This team’s confidence helps it to perform at its best.” When individual performance (i.e., number of average points scored) and team performance (i.e., final team ranking) were regressed on two indices of efficacy of group, self-efficacy of group was associated with higher individual performance, whereas collective efficacy of group was associated with higher team performance [25]. The results indicate that individual-level or idiosyncratic self-efficacy was a better predictor for individual performance; whereas, group-level or collective efficacy was a better predictor for group performance. Inferring from these results, we can reasonably expect greater predictive validity by using shared relationship efficacy of dyad, in addition to idiosyncratic relationship efficacy of dyad, as predictors in the current study.

Taking part in close relationships that are characterized by a high shared relationship efficacy of dyad is known to result in positive outcomes for people. For example, Asano [11] reported that shared relationship efficacy of dyad was associated with an increase in positive affect among romantic partners. In addition, collective efficacy of neighborhood was associated with a series of positive outcomes such as an increase in mental and physical health in the neighborhood [26]. Based on these findings, we expected that higher shared relationship efficacy of dyad would predict levels of global life satisfaction. Life satisfaction is a cognitive appraisal about one’s life, and people have higher life satisfaction when they experience positive events and avoid negative events [27,28]. Past research showed that life satisfaction is linked to important outcomes in modern societies, such as income, political actions, and educational attainment [29,30].

Although these findings received interest from many researchers and acquired supportive evidence, Oishi et al. [31] encouraged researchers to investigate characteristics of the relationship itself to predict life satisfaction, in addition to idiosyncratic characteristics of each person. To the best of our knowledge, the current study is the first to systematically examine the dyad-level processes from characteristics of the relationship itself that lead to life satisfaction.

### Hypotheses and Overview

Based on the above discussions, we hypothesized that within a relationship, shared relationship efficacy of dyad would predict life satisfaction. To test this hypothesis, we conducted two studies. In Study 1, we recruited pairs of close same-sex friends to find correlational support for the hypothesis using a cross-sectional design. In Study 2, we recruited romantic heterosexual couples to establish the causality with two-wave longitudinal data (2-month interval between Time 1 and Time 2).

## Study 1

### Materials and Methods

#### Ethical Statement

The protocols of the studies were approved by the Research Ethics Committee of the Graduate School of Education and Human Development, Nagoya University. All participants were given a description of the research and provided written informed consent for their participation.

#### Participants

A total of 137 pairs of close same-sex friends (27 males and 110 females) from five universities (Aichi Shukutoku University, Chukyo University, Kinjo Gakuin University, Meijo University, and Nagoya Women’s University) in Nagoya, Japan, participated in Study 1. At least one friend of each pair expressed an interest in participating in the study to receive partial course credit in an introductory psychology course. The mean age of males was 19.5 years (*SD* = 0.79), and the mean age of females was 19.7 years (*SD* = 1.08). Each pair had been friends for an average of 49.0 months (ranging from 4 months to 240 months).

#### Procedure

All participants received questionnaire booklets and were asked to independently complete the questionnaire at home without discussing their answers with their counterparts. Participants sealed their completed questionnaire in an envelope provided by the researchers and handed in the envelopes after 1 or 2 weeks. Upon returning the questionnaire, each person in a pair received book coupons worth 500 yen (about $4.20) for their participation.

#### Measures

*Relationship efficacy of dyad* was assessed using a modified and abbreviated nine-item version of Murray and Holmes’s [17] scale for efficacy expectations regarding close relationships. The questionnaire had been translated into Japanese and validated by Asano [32]. Examples of items include “We can successfully work through any incompatibilities between our needs,” and “We possess the communication and problem solving skills necessary to successfully resolve all of our differences.” Participants reported the degree to which each statement applied to their relationship (1 = strongly disagree to 5 = strongly agree). Responses were averaged to yield the relationship efficacy of dyad score of each friend; higher scores indicated greater levels of relationship efficacy of dyad (M = 3.68, SD = 0.58, range = 1.33–5.00, ω = .89).

*Life satisfaction* was measured using Oishi’s [33] translation of the Satisfaction With Life Scale (SWLS; [34]), which is a well-validated measure with five items. Examples of items include “In most ways my life is close to my ideal,” and “The conditions of my life are excellent.” Participants reported the degree to which each statement applied to them (1 = *strongly disagree* to 7 = *strongly agree*). Responses were summed to create a life satisfaction score for each friend; higher scores indicated greater levels of life satisfaction (*M* = 17.89, *SD* = 6.08, range = 5–30, ω = .89).

In addition, we used a modified Relationship Closeness Inventory (RCI; [35]), translated into Japanese by Kubo [36], to measure three properties of relationship interdependence, namely *frequency* of time together, *diversity* of activity, and *strength* of the influence of friend, as control variables. We included these variables because life satisfaction can be higher when the two people established interdependence [37]. By controlling for the effects of these three properties of relationship interdependence, we aimed to show whether shared relationship efficacy of dyad explained variance of life satisfaction above and beyond relationship interdependence between close friends.

#### Data Analysis

Most studies on close relationships have employed the actor—partner interdependence model (APIM; [38]), which estimates the intrapersonal effect of a person’s predictor variable on his or her own outcome variable (actor effect) and the interpersonal effect of a person’s predictor variable on the partner’s outcome (partner effect). For example, the APIM provides estimates of a person’s idiosyncratic relationship efficacy of dyad on his or her own life satisfaction, as well as the partner’s life satisfaction. However, the APIM does not allow us to treat characteristics of a relationship itself, because the analysis is not based on how the responses from one member of a dyad are similar to those of the other member of the dyad [39,40].

As noted above, our theoretical assumption is that the dyad-level effect of shared relationship efficacy of dyad on life satisfaction should be separately analyzed from the individual-level effect of idiosyncratic relationship efficacy of dyad on life satisfaction. Because we recruited pairs of close same-sex friends, their responses were not independent from each other, and thus the data violated the independence assumption of popular inferential statistics, such as analysis of variance or multiple regression analysis. In order to circumvent the nonindependence of the data, we used multilevel structural equation modeling (MSEM; [41]) to test the effects of shared and idiosyncratic relationship efficacy of dyad on life satisfaction. The MSEM decomposed the total variance of assessed variables, which were measured by individual responses within each dyad (cluster), into two orthogonal latent components, namely *between* and *within* models. The between model and the within model include shared variances between two individuals (i.e., dyad level) and unique variances of each person (i.e., individual level), respectively. In other words, the between model represents the effect of shared relationship efficacy of dyad on life satisfaction, while the within model represents the effect of idiosyncratic relationship efficacy of dyad on life satisfaction.

The MSEM has two advantages compared to a standard multilevel modeling (MLM; [42]) when we examine the shared relationship efficacy of dyad as a characteristic of the relationship itself [15]. First, the between model of the MSEM is based on reflective aggregations which infer unobserved latent dyad average, whereas the upper level (level 2) of the MLM is based on formative aggregations which represent observed dyad average. That is, in contrast to MLM, MSEM derives the average of a pair based on shared variance in their scores. Such a method is consistent with the conceptualization of shared relationship efficacy of dyad [43], because it represents an emergent property of a relationship that could be more than the simple sum of two individuals. Second, the MSEM in the between model addresses the issue that the MLM at the level 2 tends to produce biased estimates and small standard errors of coefficients, when the number of observations per cluster and number of clusters are small and the ICCs are low [43,44]. Given that the number of observations per cluster is only two for the current study and the number of dyads are relatively small, the MSEM is suitable method to operationalize shared relationship efficacy of dyad.

Following Kenny and La Voie’s [45] method, we calculated intraclass correlations (ICCs) and correlations among the assessed variables at the dyad and individual levels. These values represent the degree of similarity between two individuals in answering each questionnaire, and were used to separately examine the dyad- and individual-level processes. Then, we tested our hypothesis using the MSEM with full information maximum likelihood robust (MLR) estimation to deal with missing values and obtain standard errors that are robust to nonnormality of the variables. We included gender, relationship duration, frequency, diversity, and strength as control variables in the analysis. These statistical analyses were performed using Mplus 7.4 [46] (see S1 Appendix for the Mplus syntax).

### Results

#### Preliminary Analyses

Table 1 shows the ICCs and dyad- and individual-level correlations among the measured variables. The ICC for the relationship efficacy of dyad indicated that the similarity between friends in a pair explained 45% of total variance, suggesting that relationship efficacy of dyad is indeed shared between friends, albeit imperfectly. The ICC for life satisfaction also indicated the similarity between friends in a pair (13% of total variance). These results suggest that the MSEM is an appropriate statistical method for the current study [43,44].

**Table 1:** Intraclass and Dyad- and Individual-Level Correlations between Variables among Close Same-Sex Friendships in Study 1 (*N* = 137 pairs).

	1		2		3		4		5		6		7	
1. Relationship efficacy of dyad	**.45**	***	.14		.24	**	−.13		.20	*	.44	***	.73	**
2. Gender	.00		**1**	***	.16		−.06		.05		−.05		−.35	
3. Relationship duration	.00		.00		**1**	***	−.55	***	.03		.02		−.02	
4. Frequency	.14	*	.00		.00		**.86**	***	.27	**	.41	***	.04	
5. Diversity	.03		.00		.00		.05		**.86**	***	.50	***	−.18	
6. Strength	.29	***	.00		.00		.24	***	.12	*	**.54**	***	.29	
7. Life satisfaction	.21	**	.00		.00		−.05		.13		.05		**.13**	

*Note*. Boldface values are the intraclass correlations. Values above the diagonal are dyad-level correlations; values below the diagonal are individual-level correlations.

* *p* < .05,

** *p* < .01,

*** *p* < .001.

In addition, we found a significant positive correlation between relationship efficacy of dyad and life satisfaction at the dyad level, *r* = .727, *p* = .001, indicating that pairs of participants reported higher life satisfaction as they reported a higher degree of shared relationship efficacy of dyad. Finally, at the individual level, we found a significant positive correlation between relationship efficacy of dyad and life satisfaction, *r* = .209, *p* = .003, suggesting that participants reported higher life satisfaction as they held a higher degree of idiosyncratic relationship efficacy of dyad.

#### Relationship Efficacy of Dyad and Life Satisfaction

We conducted the MSEM to examine whether relationship efficacy of dyad was associated with life satisfaction in the between and within models. Because the model was saturated, the fit indices were perfect, χ^2^ (*df* = 0, *N* = 137 pairs) = 0.00, *p* = 1.000. As seen in Table 2, the results showed that relationship efficacy of dyad was positively associated with life satisfaction in the between model, even after controlling for gender, relationship duration, frequency, diversity, and strength, *B* = 5.441, 95% CI [2.878, 8.003], *p* < .001, β = .922. Because the between model included shared variances between two individuals (dyad level), our finding indicated that pairs of friends reported higher life satisfaction as they reported a higher degree of shared relationship efficacy of dyad. This effect of shared relationship efficacy of dyad was above and beyond the effect of gender, relationship duration, and the three properties of relationship interdependence. Relationship efficacy of dyad was also positively associated with life satisfaction in the within model, even after controlling for frequency, diversity, and strength, *B* = 2.901, 95% CI [0.883, 4.919], *p* = .005, β = .219. Because the within model included unique variances of each person (individual level), our finding demonstrated that each participant reported higher life satisfaction as he or she reported higher degree of idiosyncratic relationship efficacy of dyad. This effect of idiosyncratic relationship efficacy of dyad was above and beyond the effect of the three properties of relationship interdependence. For both the between and within models, excluding control variables did not change the pattern of results, *B* = 4.302, 95% CI [1.940, 6.664], *p* < .001, β = .762; and *B* = 2.751, 95% CI [0.796, 4.706], *p* = .006, β = .308, respectively. Therefore, as expected, shared and idiosyncratic relationship efficacy of dyad predicted higher life satisfaction.

**Table 2:** Multilevel Stractural Equation Model Predicting Life Satisfaction among Close Same-Sex Friendships in Study 1 (*N* = 137 pairs).

	*B*	95% CI	*p*	β
Between model				
Relationship efficacy of dyad	5.441	[2.878, 8.003]	< .001	.922
Gender	−2.623	[−4.618, −0.629]	.010	−.439
Relationship duration	−0.060	[−0.680, 0.559]	.849	−.037
Frequency	0.340	[−0.513, 1.193]	.435	.233
Divesity	−0.465	[−0.924, −0.007]	.047	−.383
Strength	−0.114	[−1.841, 1.614]	.897	−.045
Within model				
Relationship efficacy of dyad	2.901	[0.883, 4.919]	.005	.219
Frequency	−0.754	[−2.376, 0.867]	.362	−.083
Diversity	0.892	[−0.207, 1.992]	.112	.130
Strength	−0.066	[−1.087, 0.954]	.898	−.010

### Discussion

We investigated whether shared or idiosyncratic relationship efficacy of dyad was associated with levels of life satisfaction among close same-sex friends. The results of the MSEM showed that both shared and idiosyncratic relationship efficacy of dyad predicted higher life satisfaction. Although the findings from Study 1 are novel and intriguing, there are some shortcomings. First, the generalizability and robustness are limited because the sample consisted only of pairs of same-sex friends. Previous research has demonstrated that romantic heterosexual relationships are closer and more important in life than same-sex friendships [47,48]. Therefore, it is possible that the effect of shared relationship efficacy of dyad on life satisfaction among romantic couples could be different from that of pairs of close same-sex friends. Second, due to the correlational nature of the data in Study 1, the causal link between relationship efficacy of dyad and life satisfaction is unclear. Critics may argue that couples with higher life satisfaction meet each other frequently, partake in diverse activity, and influence each other, and as a result, develop higher levels of shared relationship efficacy of dyad.

To address these issues, we conducted Study 2, which was a two-wave longitudinal survey among romantic heterosexual couples. Study 2 had two purposes. First, we wanted to test the generalizability of the Study 1 results by targeting a different type of close dyadic relationship, namely romantic heterosexual couples. Second, in Study 2 we measured life satisfaction 2 months after measuring the relationship efficacy of dyad, which allowed us to make a causal inference about whether high shared and idiosyncratic relationship efficacy of dyad resulted in greater life satisfaction.

## Study 2

### Materials and Methods

#### Participants

We originally recruited 114 romantic heterosexual couples from four universities (Nagoya University, Kinjo Gakuin University, Meijo University, and Nagoya Women’s University) in Nagoya, Japan. At least one partner of each couple expressed an interest in participating in the research to receive partial course credit in an introductory psychology course. Among these romantic couples, 77 couples (67.5%) who completed questionnaires at both Time 1 and Time 2 were included in the following analyses. Following Srivastava and Beer’s [49] work, we conducted attrition analyses between participants who completed the study and those who dropped out. The two groups did not differ from each other in terms of assessed variables: relationship efficacy of dyad (*p* = .123, *d* = .22), relationship duration (*p* = .914, *d* = .01), frequency (*p* = .197, *d* = .18), diversity (*p* = .838, *d* = .03), and strength (*p* = .878, *d* = .02). Therefore, we decided to proceed with analysis using the remaining 77 couples. For the final set of participants, the mean age of the males was 19.9 years (*SD* = 2.31), and the mean age of the females was 19.3 years (*SD* = 1.20). The couples had been romantically involved on average for 13.0 months (range from 1 to 96 months).

#### Procedure

All participants at Time 1 received questionnaire booklets containing questions about relationship efficacy of dyad and the three properties of relationship interdependence. Participants were asked to independently fill out the questionnaires at home without discussing with their romantic partner. Participants independently sealed their completed questionnaire in an envelope provided by the researchers and handed in the envelopes after 1 or 2 weeks. Approximately 2 months later (Time 2), participants completed another booklet containing questions about life satisfaction, without discussing with their partner. They independently sealed and returned envelopes. When the couples had completed both questionnaires, they received book coupons worth 2,000 yen (about $16.7) per person for their participation.

#### Measures

We used the same scales as in Study 1. At time 1, *relationship efficacy of dyad* was assessed using Murray and Holmes’s [17] scale for efficacy expectations regarding close relationships, translated into Japanese [32]. Responses were averaged to yield a relationship efficacy of dyad score of each partner (*M* = 3.73, *SD* = 0.62, range = 1.78–5.00, ω = .89). In addition to relationship efficacy of dyad, we measured *frequency*, *diversity*, and *strength* of relationship interdependence using the RCI [35], translated into Japanese by Kubo [36]. At time 2, life satisfaction was measured using Oishi’s [33] translation of the SWLS [34]. Responses were summed to create the life satisfaction score of each partner (*M* = 20.24, *SD* = 6.86, range = 5–35, ω = .93).

#### Data Analysis

Following Study 1, we calculated the ICCs and dyad- and individual-level correlations among the assessed variables, and then performed the MSEM with full information MLR to test our hypothesis. We included gender, relationship duration, frequency, diversity, and strength as control variables. Mplus 7.4 [46] (see S2 Appendix for the Mplus syntax) was used for these statistical analyses.

### Results

#### Preliminary Analyses

Table 3 shows the ICCs and dyad- and individual-level correlations among the measured variables. The ICC for relationship efficacy of dyad indicated that the similarity between romantically involved partners in a couple explained 40% of total variance, suggesting that relationship efficacy of dyad is moderately shared between romantic partners. The ICC for life satisfaction also indicated the similarity between romantic partners in a couple (34% of total variance). These results suggest that the MSEM is an appropriate statistical method for the current study [43,44].

**Table 3:** Intraclass and Dyad- and Individual-Level Correlations between Variables among Romantic Heterosexual Relationships in Study 2 (*N* = 76–114 couples).

	1		2		3		4		5		6		7	
1. Relationship efficacy of dyad	**.40**	***	.00		.02		.28	**	.16		.33	**	.55	**
2. Gender	−.07		−**1**	***	.00		.00		.00		.00		.00	
3. Relationship duration	.00		.00		**1**	***	.07		−.06		−.03		−.03	
4. Frequency	−.12		.05		.00		**.81**	***	.33	**	.52	***	−.26	
5. Diversity	−.10		.03		.00		−.03		**.75**	***	.78	***	−.06	
6. Strength	.19		.11		.00		.22	**	.05		**.38**	***	−.09	
7. Life satisfaction	.24	*	−.06		.00		.27	*	.08		.26	*	**.34**	**

*Note*. Boldface values are the intraclass correlations. Values above the diagonal are dyad-level correlations; values below the diagonal are individual-level correlations.

* *p* < .05,

** *p* < .01,

*** *p* < .001.

We also found a significant positive correlation between relationship efficacy of dyad and life satisfaction at the dyad level, *r* = .553, *p* = .001, indicating that couples reported higher life satisfaction as they reported a higher degree of shared relationship efficacy of dyad. Finally, at the individual level, we found a significant positive correlation between relationship efficacy of dyad and life satisfaction, *r* = .242, *p* = .031, suggesting that participants’ life satisfaction was higher as they held higher degrees of idiosyncratic relationship efficacy of dyad.

#### Relationship Efficacy of Dyad and Life Satisfaction

We conducted the MSEM to investigate whether initial relationship efficacy of dyad (Time 1) was associated with life satisfaction at a later time (Time 2) in the between and within models. The between model and the within model included shared variances between two individuals (i.e., dyad level) and unique variances of each person (i.e., individual level), respectively. The between model represents the effect of shared relationship efficacy of dyad on life satisfaction, while the within model represents the effect of idiosyncratic relationship efficacy of dyad on life satisfaction. The fit indices were perfect because the model was saturated, χ^2^ (*df* = 0, *N* = 77 couples) = 0.00, *p* = 1.000. As seen in Table 4, the results showed that relationship efficacy of dyad was positively associated with life satisfaction in the between model, even after controlling for relationship duration, frequency, diversity, and strength, *B* = 6.552, 95% CI [2.499, 10.605], *p* = .002, β = .713. Our finding demonstrated that both partners in a couple reported higher life satisfaction as they reported higher degrees of shared relationship efficacy of dyad. Relationship efficacy was also positively associated with life satisfaction in the within model, even after controlling for gender, frequency, diversity, and strength, *B* = 2.572, 95% CI [0.015, 5.129], *p* = .049, β = .230. Our finding demonstrated that partners reported higher life satisfaction as they reported higher degrees of idiosyncratic relationship efficacy of dyad. For both the between and within models, excluding control variables did not change the pattern of results, *B* = 4.961, 95% CI [1.405, 8.517], *p* = .006, β = .542; and *B* = 2.753, 95% CI [0.212, 5.294], *p* = .034, β = .246, respectively. Therefore, as expected, shared and idiosyncratic relationship efficacy of dyad predicted higher life satisfaction.

**Table 4:** Multilevel Stractural Equation Model Predicting Life Satisfaction among Romantic Heterosexual Relationships in Study 2 (*N* = 77 couples).

	*B*	95% CI	*p*	β
Between model				
Relationship efficacy of dyad	6.552	[2.499, 10.605]	.002	.713
Relationship duration	0.108	[−0.633, 0.848]	.776	.043
Frequency	−1.330	[−3.084, 0.424]	.137	−.391
Divesity	0.308	[−2.168, 2.784]	.807	.139
Strength	−1.024	[−7.730, 5.682]	.765	−.226
Within model				
Relationship efficacy of dyad	2.572	[0.015, 5.129]	.049	.230
Gender	−0.893	[−2.604, 0.817]	.306	−.080
Frequency	2.199	[0.170, 4.228]	.034	.266
Diversity	0.308	[−0.882, 1.596]	.572	.062
Strength	0.762	[−0.252, 1.776]	.141	.164

### Discussion

In Study 2, although we used a different type of relationship (i.e., romantic heterosexual couples) and study design (i.e., longitudinal data) from those used in Study 1, we successfully replicated the same pattern of results. The results of the MSEM showed that shared and idiosyncratic relationship efficacy of dyad at one point in time predicted greater life satisfaction among romantic partners 2 months later, thus supporting our hypothesis. The findings suggest the robustness of our model by showing that dyads with high shared relationship efficacy of dyad, and individuals with high idiosyncratic relationship efficacy of dyad, experience higher life satisfaction.

## General Discussion

We conducted two studies to examine whether the efficacy expectations mutually held by members in a dyadic relationship—or shared relationship efficacy of dyad—predicted their life satisfaction. In Study 1, we collected data from close same-sex friends and found that shared relationship efficacy of dyad was associated with a higher levels of life satisfaction. In Study 2, we recruited romantic heterosexual couples and measured shared relationship efficacy of dyad 2 months before the measurement of life satisfaction. In this longitudinal study, levels of shared relationship efficacy of dyad predicted levels of couples’ life satisfaction in the future. In both studies, the effect of shared relationship efficacy of dyad was above and beyond the effect of the three properties of relationship interdependence. That is, efficacy expectations shared between individuals explained additional variance in life satisfaction beyond the effects of the actual interaction patterns within a dyad. Accumulated evidence suggests that strong and stable bonds between individuals maintain and/or enhance life satisfaction [50,51]. However, characteristics of the relationship itself have rarely been used for predicting levels of life satisfaction [10] (see also [52]). As Oishi et al. [31] pointed out, the prevailing focus on individual-level variables, and failure to take into account phenomena at the dyad or collective level, holds back the development of research. The current study offered a new way to empirically investigate the effects of the relationship as a whole on life satisfaction, and could stimulate future research regarding close relationships and life satisfaction.

Although our findings can be a stepping stone for other close relationship research on dyad-level processes, the underlying mechanisms are still unclear. We speculate that resource allocation to a relationship may mediate the effect of shared relationship efficacy of dyad on life satisfaction. That is, compared to close friendships or romantic couples with a low degree of shared relationship efficacy of dyad, those with high degrees of shared relationship efficacy might invest more financial, physical, or psychological resources in their relationships and create a social environment conducive to a harmonious relationship. As a result, they might experience greater levels of life satisfaction. Supporting this view, Zaccaro et al. [53] argued that collective efficacy results in positive outcomes when every group member contributes their resources, such as knowledge, skills, or abilities, to accomplish goals.

At the individual-level processes, our findings for idiosyncratic relationship efficacy shed light on underlying mechanisms. We found that idiosyncratic relationship efficacy of dyad was associated with higher life satisfaction among close same-sex friends and among romantic partners. In the past, Murray and Holmes [17] reported that romantic or marital couples who hold an unusually high degree of idiosyncratic relationship efficacy of dyad were likely to report high relationship quality in terms of satisfaction, trust, and long-term stability in the relationship. Based on these two findings, we can reasonably speculate that idiosyncratic relationship efficacy of dyad, life satisfaction, and relationship qualities are interrelated with each other. Because we found that idiosyncratic relationship efficacy of dyad results in higher life satisfaction, future research should identify the directional relationship between relationship quality and life satisfaction to elucidate the impact of idiosyncratic relationship efficacy of dyad in close relationships.

Several limitations need to be addressed in future research. The first is the generalizability of our findings, as the age range for dyads was restricted to young adults. We targeted this population because the years spent at college and university often represent a time in the life cycle when individuals are developing long-term relationships with others. Future research will need to investigate whether the pattern of results could be replicated in other social relationships such as marital relationships, parent—child relationships, or work relationships. Second, a limitation of Study 2 is the failure to measure changes in levels of life satisfaction between Time 1 and Time 2. Although it is theoretically meaningful to investigate the increase or decrease of life satisfaction, a main purpose of these two studies was to test whether the characteristics of the relationship itself can predict positive outcomes in close friendships (Study 1) or romantic relationships (Study 2). To this end, we followed the design of other longitudinal studies in social and personality psychology (e.g., [54,55]). Third, future research might benefit by adding more outcome variables for well-being. Although we assessed only global life satisfaction as an outcome variable to avoid participants’ fatigue, measures for specific types of well-being such as hedonia (e.g., positive or negative affect) or eudaimonia (e.g., personal growth, purpose in life, and autonomy) would allow researchers to elucidate the specific nature of well-being [56,57].

Despite these limitations, this is the first evidence to show that shared relationship efficacy of dyad, as well as idiosyncratic relationship efficacy of dyad, was associated with higher life satisfaction among close same-sex friends and romantic heterosexual partners. Most studies on close relationships and life satisfaction have overlooked the dyad-level processes resulting from the characteristics of the relationship itself, including shared relationship efficacy of dyad, although it is recognized that such characteristics should be empirically investigated. Our findings provide one way to overcome the shortcomings in the literature. We argue that the effects of close relationships on life satisfaction cannot be understood unless the pivotal role of shared relationship efficacy of dyad is fully taken into account.

## Supporting Information

S1 Appendix Mplus Syntax for the Multilevel Structural Equation Modeling in Study 1. (DOCX)

S2 Appendix Mplus Syntax for the Multilevel Structural Equation Modeling in Study 2. (DOCX)
