# Correction: Shared Relationship Efficacy of Dyad Can Increase Life Satisfaction in Close Relationships: Multilevel Study

**DOI:** 10.1371/journal.pone.0167688

**Published:** 2016-12-01

**Authors:** Ryosuke Asano, Kenichi Ito, Toshikazu Yoshida

There is information missing from the Funding section. The correct funding information is as follows: This research was supported by JSPS Research Fellowships for Young Scientists (23–2815) and special funding for the promotion of internationalization of research activities by the Japanese Group Dynamics Association to RA. The funder had no role in study design, data collection and analysis, decision to publish, or preparation of the manuscript.
