# Supplementary material for: Shared Relationship Efficacy of Dyad Can Increase Life Satisfaction in Close Relationships: Multilevel Study
Source: PLoS One. 2016 Jul 20;11(7):e0159822. doi: 10.1371/journal.pone.0159822 (PMC4954727; doi:10.1371/journal.pone.0159822)
Supplement: S1 Appendix — (DOCX) [file pone.0159822.s001.docx]

**S1 Appendix. Mplus Syntax for the Multilevel Structural Equation Modeling in Study 1**

TITLE: MSEM among Friendships in Study 1

DATA: FILE IS Friend_Study1.csv;

VARIABLE: NAMES = pair gender durat freq dive stre reeffi lifesa;

MISSING = .; !identify missing value

CLUSTER = pair; !identify Level 2 grouping variable

BETWEEN = gender durat; !identify variables at Level 2

ANALYSIS: TYPE = TWOLEVEL;

MODEL: %WITHIN%

lifesa ON reeffi freq dive stre;

!life satisfaction regressed on relationship efficacy of dyad controlling for the properties of relationship interdependence at the individual level

%BETWEEN%

lifesa ON reeffi gender durat freq dive stre;

!life satisfaction regressed on relationship efficacy of dyad controlling for gender, relationship duration, and the properties of relationship interdependence at the dyad level
