# Supplementary material for: Shared Relationship Efficacy of Dyad Can Increase Life Satisfaction in Close Relationships: Multilevel Study
Source: PLoS One. 2016 Jul 20;11(7):e0159822. doi: 10.1371/journal.pone.0159822 (PMC4954727; doi:10.1371/journal.pone.0159822)
Supplement: S2 Appendix — (DOCX) [file pone.0159822.s002.docx]

**S2 Appendix. Mplus Syntax for the Multilevel Structural Equation Modeling in Study 2**

TITLE: MSEM among Romantic Relationships in Study 2

DATA: FILE IS Couple_Study2.csv;

VARIABLE: NAMES = pair gender durat freq dive stre reeffi lifesa;

MISSING = .; !identify missing value

CLUSTER = pair; !identify Level 2 grouping variable

WITHIN = gender; !identify variable at Level 1

BETWEEN = durat; !identify variable at Level 2

ANALYSIS: TYPE = TWOLEVEL;

MODEL: %WITHIN%

lifesa ON reeffi gender freq dive stre;

!life satisfaction regressed on relationship efficacy of dyad controlling for gender and the properties of relationship interdependence at the individual level

%BETWEEN%

lifesa ON reeffi durat freq dive stre;

!life satisfaction regressed on relationship efficacy of dyad controlling for relationship duration and the properties of relationship interdependence at the dyad level
